# Supplementary material for: CoFeS2@CoS2 Nanocubes Entangled with CNT for Efficient Bifunctional Performance for Oxygen Evolution and Oxygen Reduction Reactions
Source: Nanomaterials (Basel). 2022 Mar 16;12(6):983. doi: 10.3390/nano12060983 (PMC8952201; doi:10.3390/nano12060983)
Supplement: Supplementary file 1 [file nanomaterials-12-00983-s001.zip › nanomaterials-1633803-supplementary.pdf]

nanomaterials

MDPI

Supplementary Material

$CoFeS_2@CoS_2$ Nanocubes Entangled with CNT for Efficient Bifunctional Performance for Oxygen Evolution and Oxygen Reduction Reactions

Jaeeun Jeon [1,†], Kyoung Ryeol Park [2,†], Kang Min Kim [3,†], Daehyeon Ko [4], HyukSu Han [5], Nuri Oh [6], Sunghwan Yeo [7,*], Chisung Ahn [1,*] and Sungwook Mhin [4,*]

1 Korea Institute of Industrial Technology, 113-58, Seohaean-ro, Siheung 15014, Republic of Korea; jaeeun00@kitech.re.kr
2 Korea Institute of Industrial Technology, 55 Jongga-ro, Jung-gu, Ulsan 44413, Republic of Korea; krpark@kitech.re.kr
3 Korea Institute of Industrial Technology, 137-41 Gwahakdanji-ro, Gangneung 25440, Republic of Korea; kmkim@kitech.re.kr
4 Department of Advanced Materials Engineering, Kyonggi University, Suwon 16227, Republic of Korea; godh3134@naver.com
5 Department of Energy Engineering, Konkuk University, Seoul 05029, Republic of Korea; hhan@konkuk.ac.kr
6 Department of Advanced Materials Engineering, Hanyang University, Seoul 04763, Republic of Korea; irunho@hanyang.ac.kr
7 Korea Atomic Energy Research Institute, Daedeok-Daero 989-111, Yuseong-gu, Deajeon 305353, Republic of Korea
* Correspondence: syeo@kaeri.re.kr (S.Y.); cahn@kitech.re.kr (C.A.); swmhin@kgu.ac.kr (S.M.)
† These authors contributed equally to this work.

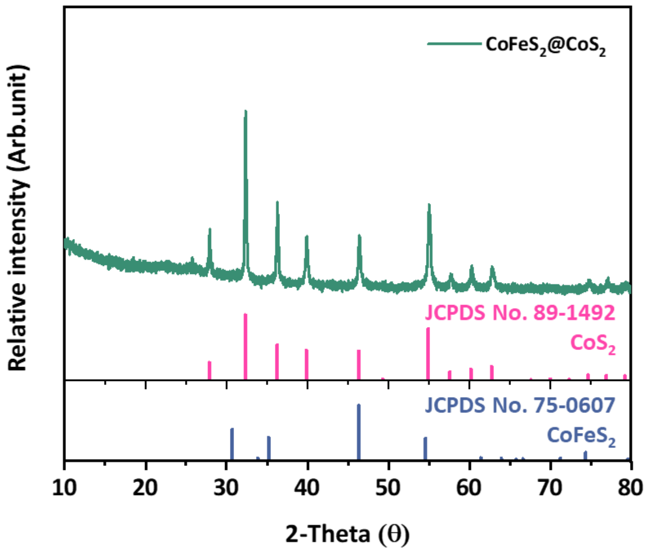

Figure S1. XRD data of pure $CoFeS_2@CoS_2$.

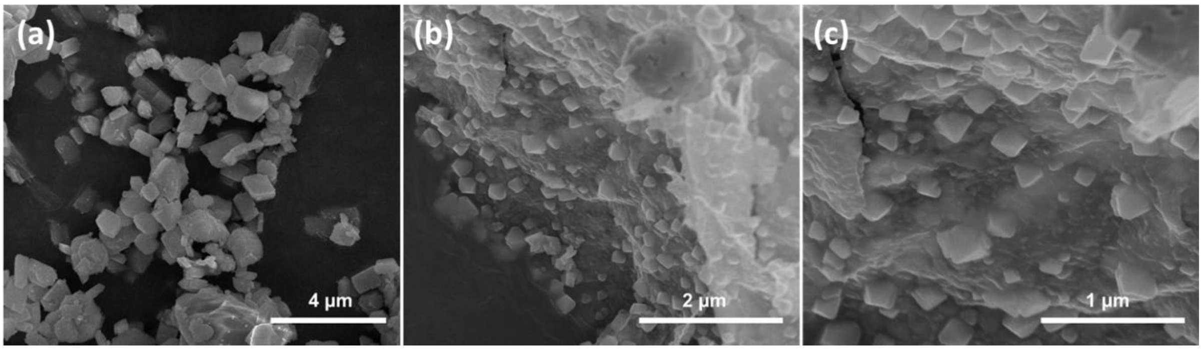

Figure S2. (**a**–**c**) Microstructure of pure $CoFeS_2@CoS_2$.

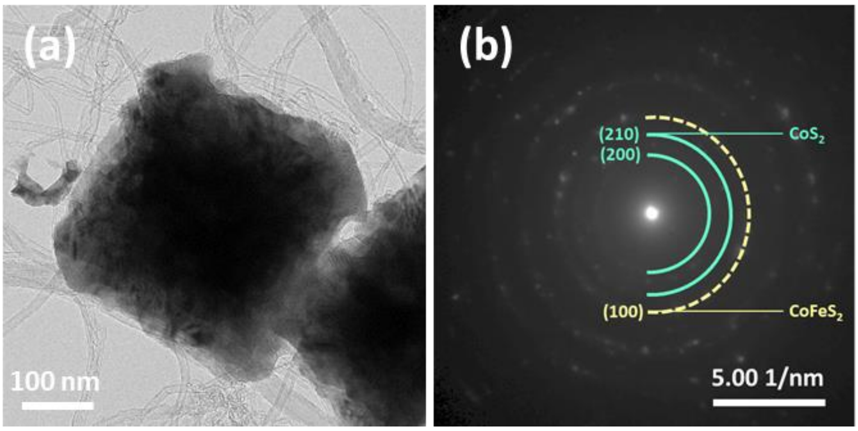

Figure S3. (**a**) TEM image of $CoFeS_2@CoS_2$/CNTs with (**b**) SAED pattern.

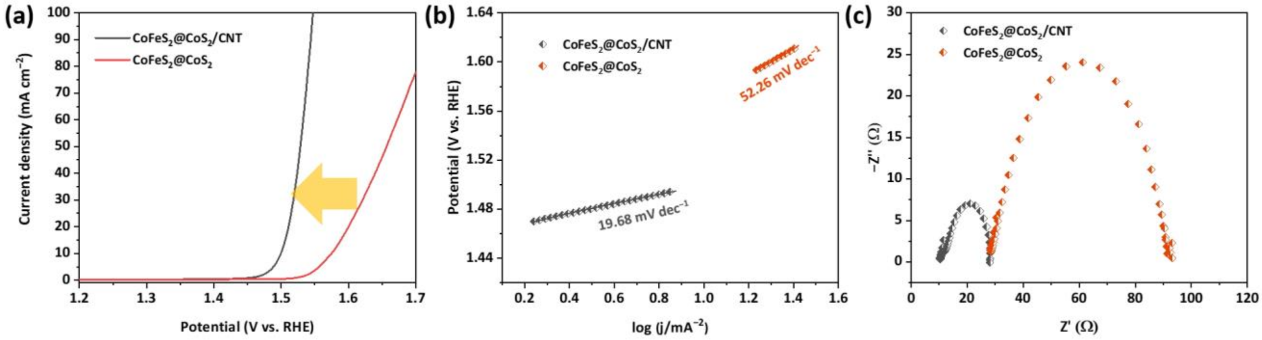

Figure S4. OER properties of the $CoFeS_2@CoS_2$ with/without CNTs (**a**) LSV curve, (**b**) Tafel plot, (**c**) Nyquist plot.

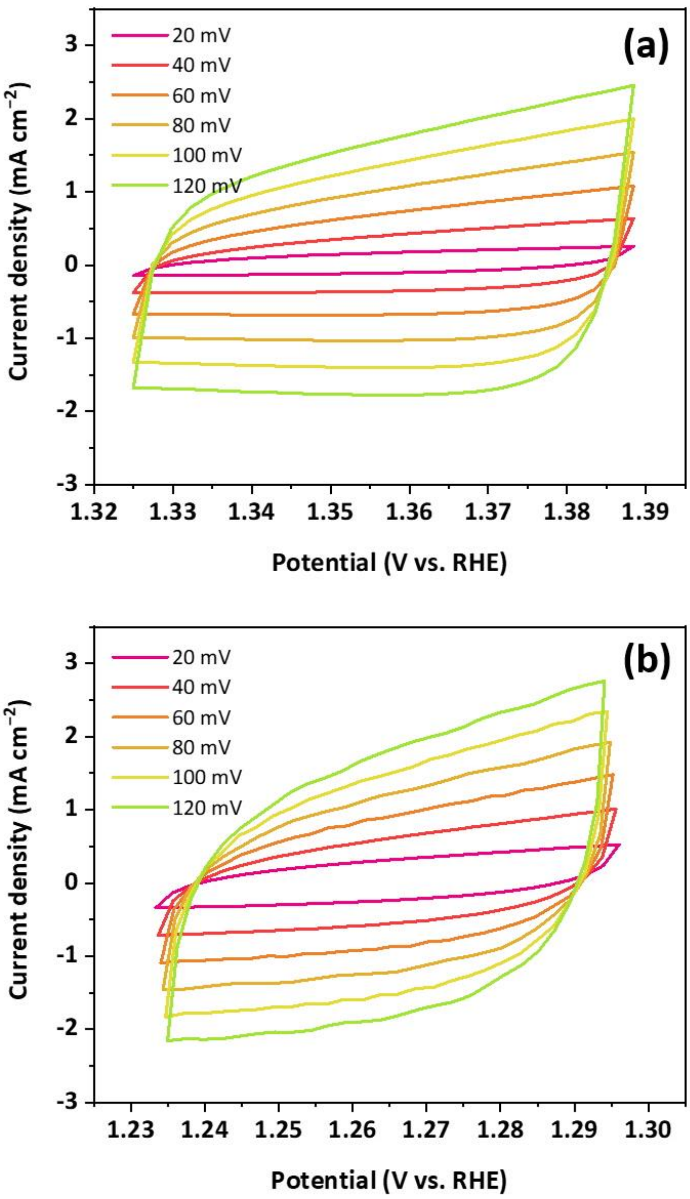

Figure S5. CV measurements in a capacitive current region at scan rates 20, 40, 60, 80, 100, and 120 s^{-1} of (**a**) CoS_2/CNTs and (**b**) $CoFeS_2$@CoS_2/CNTs.

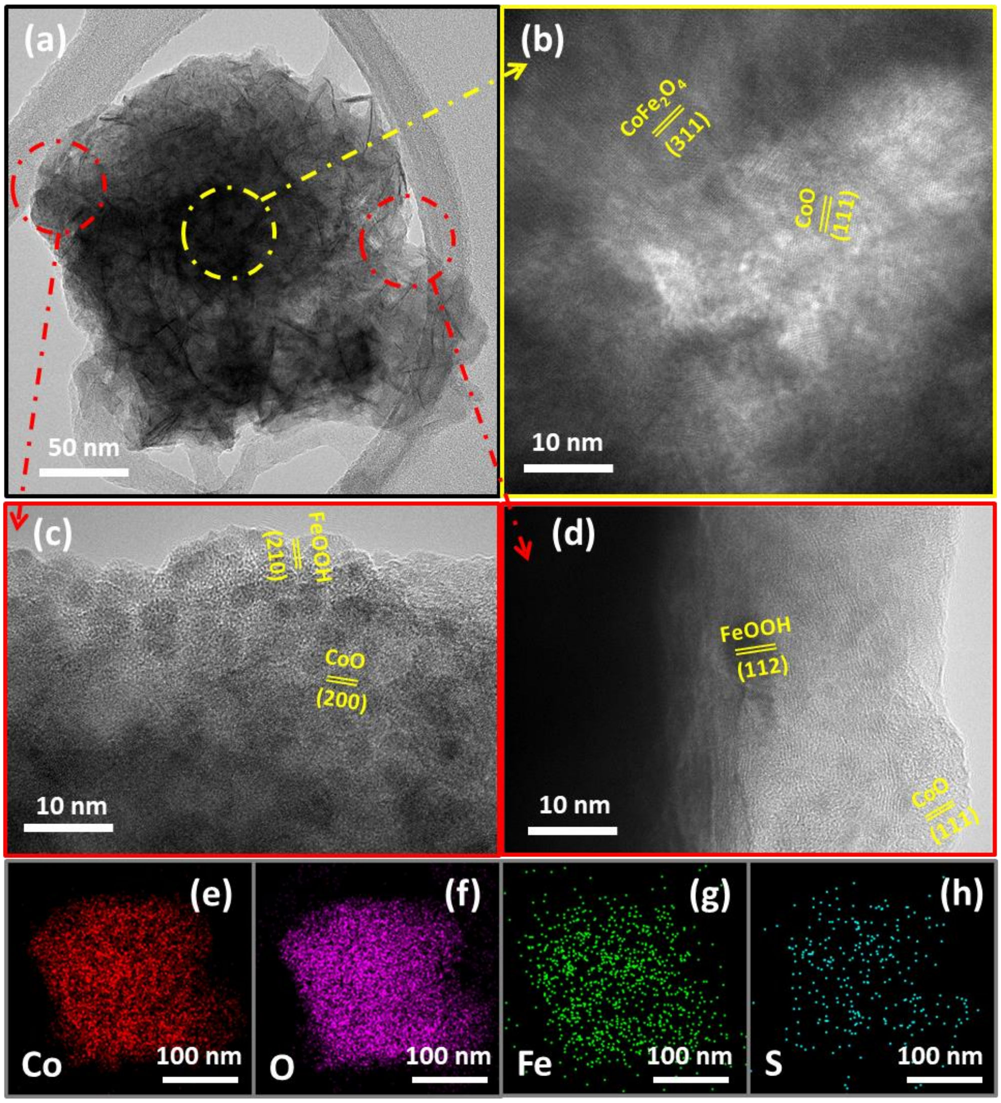

Figure S6. Morphology and structure characterizations of $CoFeS_2$@CoS_2/CNTs after stability test for 12 h. (**a**–**d**) TEM image and HRTEM images and (**e**–**h**) element maps.

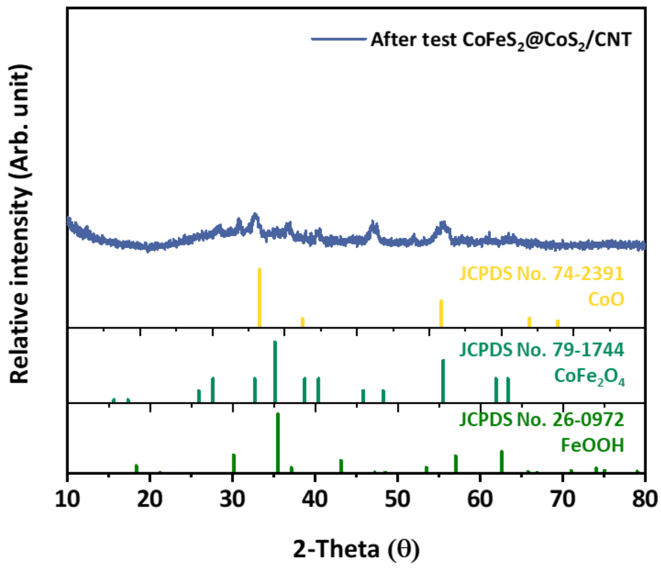

Figure S7. X-ray diffraction patterns of the $CoFeS_2@CoS_2$/CNTs after 12 h of OER.

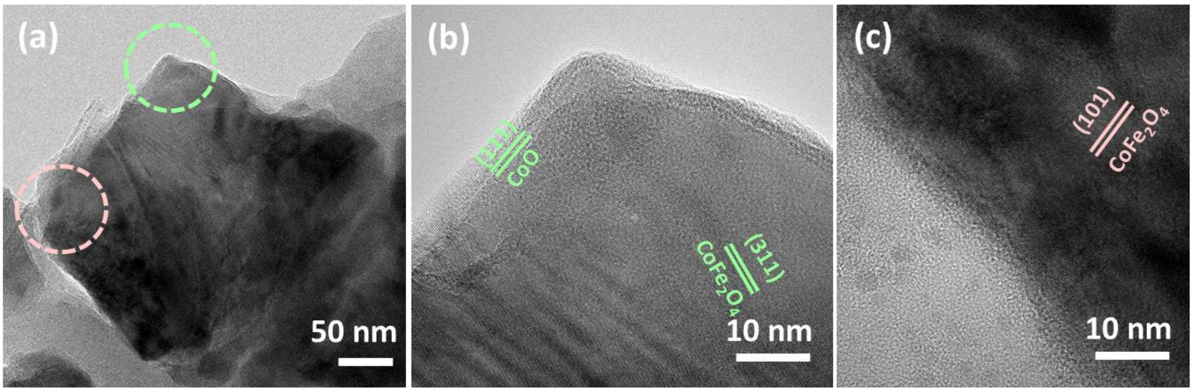

Figure S8. (**a**) TEM and (**b**–**c**) HRTEM images of the $CoFeS_2@CoS_2$/CNTs after 2000 cycles of ORR.

Table S1. Comparison of OER/ORR catalytic activity of $CoFeS_2@CoS_2$/CNTs with the recently reported Co, Fe-based catalysts.

Electrocatalyst	Electrolyte	Overpotential (@10 mA cm^{-2})	ORR Onset potential	Tafel slope (mV dec^{-1})	Stability	Reference
This work	1.0 M KOH	269	0.976	19.68	12 h	
CoS_2 microbox	1.0 M KOH	308	-	41.40	1000 cycled	S1
	0.5 M H_2SO_4	278	-	73.00	1000 cycled	S2

Co-FeS_2/CoS_2						
Fe_7S_8 bulk	1.0 M KOH	380	-	96.00	-	S3
NiCo-Phi/CNT	0.1 M KOH	400	0.75	117	20 h	S4

References

1. Hua, Y.; Jiang, H.; Jiang, H.; Zhang, H.; Li, C. Hierarchical porous CoS2 microboxes for efficient oxygen evolution reaction. *Electrochim. Acta.* **2018**, *278*, 219–225.
2. Wang, K.; Guo, W.; Yan, S.; Song, H.; Shi, Y. Hierarchical CoFeS2/CoS2 heterostructures as a superior bifunctional electrocatalyst. *RSC adv.* **2018**, *8(50)*, 28684–28691.
3. Ni
4. Sathiskumar, C.; Alex, C.; John, N. S. Nickel cobalt phosphite nanorods decorated with carbon nanotubes as bifunctional electrocatalysts in alkaline medium with a high yield of hydrogen peroxide. *ChemElectroChem.* **2020**, *7(8)*, 1935–1942.
